# Visual impairment and employment in Norway

**DOI:** 10.1186/s12889-022-13077-0

**Published:** 2022-04-05

**Authors:** Audun Brunes, Trond Heir

**Affiliations:** 1 Division of Mental Health and Addiction, Norwegian National Unit for Sensory Loss and Mental Health, Oslo University Hospital, Oslo, Norway; 2 Section for Trauma, Catastrophes and Forced Migration - Adults and Elderly, Norwegian Centre for Violence and Traumatic Stress Studies, Oslo, Norway; 3 Institute of Clinical Medicine, Faculty of Medicine, University of Oslo, Oslo, Norway

**Keywords:** Associated factors, Blindness, Employment, Depression, Visual impairment, Quality of life, Work integration

## Abstract

**Background:**

Past studies have suggested lower employment of people with visual impairment. Reasons for this are less known. This study aimed to examine the employment rates among people with visual impairment, and its association with sociodemographic characteristics, vision-related factors, depression, and life satisfaction.

**Methods:**

This cross-sectional study included a stratified random sample of 574 working-age adults (18–67 years) who were members of the Norwegian Association of the Blind and Partially Sighted. Data were collected by telephone interviews between January and May 2017, and included information on work status, sociodemographic factors, vision-related characteristics, depression (Patient Health Questionnaire-9), and life satisfaction (Cantril’s Ladder of Life Satisfaction). Associations with employment (full-time, part-time, or self-employment) were examined using regression analyses.

**Results:**

Of the participants, 51.2% of males and 38.1% of females reported to be employed. Employment was associated with being of middle age, male gender, higher education, residing in high-income municipalities, having a moderate degree of vision loss, lower onset-age of vision loss, and having no additional impairments. Employed participants had lower levels of depression compared to others (adjusted exponentiated beta: 0.80, 95% confidence interval (CI): 0.67, 0.96). They also had a higher odds of scoring higher on life satisfaction (adjusted exponentiated beta (odds ratio): 1.85, 95% CI: 1.32, 2.59).

**Conclusions:**

Employment was lower in people with visual impairment than in the general population. Inclusion of the blind and partially sighted into the workforce could promote health and thus have socio-economic benefits.

**Supplementary Information:**

The online version contains supplementary material available at 10.1186/s12889-022-13077-0.

## Background

Visual impairment – partial or complete loss of vision – is a heterogeneous condition, affecting about 1 billion of the world’s population [1]. People with visual impairment face great barriers in performing daily life activities and in social participation, with work participation being one of the greatest challenges [2]. Despite that many wants to work, the employment rate of people with visual impairment in Westernized countries are substantially lower relative to the general population [3–6], with a gap as large as 50 percent in some countries [3]. Furthermore, visual impairment has been associated with unfavourable conditions, such as poverty [7, 8], underemployment [8], low work satisfaction [8], and early retirement from working life [9].

Not only are equal work opportunities for people with visual impairment a main political objective in Westernized societies, better access to the labour market for these people may also have economic benefits. For example, the global costs in productivity loss due to visual impairment have been estimated to $411 billion per year [3]. As the workforce ages in most Westernized countries, the prevalence of working age people with visual impairment will increase over the next decades [10]. Instead of being viewed as a burden, people with visual impairment are now more recognized as a valuable resource to handle the future challenges of an aging workforce and securing a sustainable welfare state in Westernized countries.

The work participation of people with visual impairment may be linked to the impairment itself, to individual factors, and to social or structural barriers [11]. Except for education, there is inconclusive evidence about predictors of employment in people with visual impairment [11–13]. Much of the variation could be due to the inclusion of small and non-representative samples. It could also be influenced by how employment has been assessed and categorized across the different studies. While some studies have focused on correlates of full-time employment only, others have obtained information on employment regardless of the weekly working hours.

Work has generally a positive impact on people’s health and quality of life [14]. Along with financial security, work participation may also facilitate independent living, social inclusion, regular contact with other people, having a pre-defined role in society, and a sense of human dignity [15, 16]. In previous studies, we observed higher levels of depression and lower life satisfaction in people with visual impairment compared to the general population [17, 18]. Because of this and the low employment rates in people with visual impairment, it is of interest to study the relationship between employment and outcomes of depression and life satisfaction in this population. We hypothesized that employment could protect against the development of depression and increase people’s quality of life.

Reasons for why people with visual impairment fall outside the labour market are multifactorial. It may be related to health issues but also that the labour market is poorly adapted to groups with special needs. Standing outside of the labour market does not necessarily mean being unwilling or unable to work. For example, one in five people on disability benefits in Europe state that they are able to work [19]. Insecurities about future job career and financial income could drive potential workers into seeking disability benefits or early retirement rather than register as unemployed [20]. To offer a more complete picture on the employment situation of people with visual impairment, the present study will therefore incorporate all people not being employed regardless of whether they are active work seekers or if they raise disability benefits.

To our knowledge, there is a lack of studies about associated factors and outcomes of employment that includes a large sample of people with visual impairment. Therefore, the present study had the following main aims: 1) to explore the gap in employment between people with visual impairment and the general Norwegian population, 2) to examine sociodemographic and vision-related factors associated with employment among people with visual impairment, and 3) to investigate employment and its association with depression and life satisfaction.

## Methods

### Design and participants

#### Visual impairment population

The present study is a part of a larger cross-sectional study about serious life events and mental health in people with visual impairment in Norway. In brief, a telephone survey was administered between January and May 2017, to a random sample of members from the Norwegian Association of the Blind and Partially Sighted. This is the largest organization for people with visual impairment in Norway, consisting of more than 10,000 members [21]. All participants aged 18 years or older were eligible to participate if they had a diagnosis of visual impairment and were able to speak and understand the Norwegian language. The telephone interviews were carried out by trained interviewers employed at IPSOS MMI. The interview guide included more than 120 questions, and took on average 30 min to complete.

As most members were of old age, we therefore used age-stratified random sampling to include the entire visual impairment population. First, the study population was divided into four age groups (years: 18–35, 36–50, 51–65, ≥ 66), and then we randomly surveyed members across the different age groups. A total of 234, 315, 301, and 366 members were invited to obtain a sufficient number of participants in the age groups 18–35, 36–50, 51–65, and ≥ 66 years, respectively. The sample size calculations have been described elsewhere [22]. Overall, 736 members participated (response rate: 53–67%). Of those, we excluded 162 because they were above the age of 67 years, which is the retirement age in Norway. Therefore, the total sample comprised 574 participants.

#### General population

We extracted data on employment status for males and females in the age group 20 to 67 years from the 2017 Norwegian Labour Force Survey [23]. The Norwegian Labour Force Survey is a continuous, rotating panel, telephone survey performed by Statistics Norway [24]. Each quarter a representative sample of 12,000 households are selected from the Central Population Register, and all individuals aged 15 and 74 years in the household are invited to participate. The total quarterly sample size is about 24,000 individuals. The participants are asked detailed questions about their employment status, along with background information. To provide national representative data for the general working-age population, and to correct for sampling and non-response errors, the data are weighted and presented as population totals and percentages. The weights are created by a method called one-step multiple model-calibration [24].

### Measures

#### Employment status

The employment status of people with visual impairment was determined by a single question (“What is your current employment status”), whereas for the general population it was obtained by several different questions. In the analyses including people with visual impairment, we created an employment variable and classified it into two categories: ‘*not employed*’ (0) and ‘*employed*’ (1). Participants who reported any type of paid work, including full-time, part-time, or self-employment, were classified as ‘*employed*’. The ‘*not employed*’ category encompassed participants not having paid work, including those being unemployed or outside of work force for different reasons (i.e., under education, retirement, disability benefits, home workers, and others).

#### Depression

For people with visual impairment, the nine-item Patient Health Questionnaire (PHQ-9) was applied as a measure of depression [25]. The validity and utility of the PHQ-9 are good [26], and the symptoms correspond to those listed in the *Diagnostic and Statistical Manual of Mental Disorders, fifth edition*. The participants were asked to report how much a problem had bothered them during the past two weeks, with a response ranging from ‘*not at all*’ (0) to ‘*nearly every day*’ (3). A total score was created by adding each of the nine items together, ranging from 0 to 27 points, with a higher score indicating more depressive symptoms. General population data from Norway show a mean PHQ-9 score of 2.7 for men and 3.5 for women [27]. The items had a Cronbach’s alpha of 0.84. In the main analyses, the total score was treated as an untransformed continuous variable.

#### Life satisfaction

Cantril’s Ladder of Life Satisfaction was employed in the questionnaire to measure current life satisfaction [28]. The participants were asked to imagine a ladder with 10 steps, with the bottom step representing the worst possible life (a score of 1) and the top step representing the best possible life (a score of 10). The general Norwegian population scores on average 7.6 points on this scale [29]. In our study, the scale was treated as an untransformed continuous variable in the main analyses.

#### Covariates

Based on data availability and previous publications [10–12], the following sociodemographic and vision-related characteristics were considered relevant for this study: age, gender, education (years: < 13, ≥ 13), marital status (married/cohabitant, other), municipal income level (low, medium, high), and self-reported severity of vision loss (moderate, severe/blind). We also created an ‘*onset-age of vision loss*’ variable by subtracting the participant’s age with the number of years since onset of vision loss. The variable was either treated as continuous or categorized into the following three categories: ‘*congenital*’, ‘*childhood/adolescence (1–24 years)*’, and ‘*adulthood (*≥ *25 years)*’. Lastly, the participants were asked whether they had other impairments in addition to vision loss (no, yes) and, if responding ‘*yes*’, they were then asked to report what type of conditions that they had. Frequently reported conditions included hearing loss, movement disorders, musculoskeletal pain, cardiovascular disorders, and diabetes.

### Ethics

The study was carried out in accordance with principles of anonymized data, and approved by the Regional Committee for Medical and Health Research Ethics (Reference number: 2016/1615A). No identifying information was collected. The participants were informed about all aspects of the project, including potential risks and the voluntary nature of the survey, and consented by completing the survey.

### Statistical analysis

We used Stata Version 16 (Stata Corp., Texas, USA) for all statistical analyses. The significance level was set at *p* = 0.05. Descriptive statistics were presented for males and females separately. We also compared the employment status for males and females with visual impairment against the general population, and calculated percentage difference between the two populations to estimate the employment gap. Differences in categorical variables were examined using Pearson’s chi-squared tests.

Robust log-Poission Generalized Linear Models (GLMs) [30] were performed to estimate probability ratios (PR) and 95% confidence intervals (CIs) for employment and its association with sociodemographic and vision-related factors. The independent variables were entered into one block, and included age (years: 18–35, 36–50, 51–67), gender, education (years: < 13, ≥ 13), municipal income level (low, medium, high), severity of vision loss (moderate, severe/blind), onset-age of vision loss, and having other impairments (no, yes). Because of the uncertain causal nature of marital status, we decided to exclude the variable from the analysis. Furthermore, we found a significant product term between severity and onset-age of vision loss (χ^2^: 5.1, *p* = 0.02). However, adding the product term into the adjusted model did not affect model fit, and we therefore decided to present main effects only. No other product terms reached statistical significance (each *p* > 0.05).

To examine the association between employment and outcomes of depression and life satisfaction, we applied different types of regression models. For depression, we used the log-Gamma GLM [31]. For life satisfaction, we used the heterogeneous choice model [32]. We found a violation of the proportional odds assumption for several variables (i.e., employment status, age, education, marital status, municipal income level, and onset-age of vision loss). The main advantage of using heterogeneous choice models in such situations instead of ordinal logistic regression is that we can specify in the model the variables that violates the proportionality assumption, and thus correct for it [32]. The regression models were either crude (unadjusted) or adjusted for all indicated covariates. The results were presented as exponentiated beta-values and 95% CIs. For the gamma GLM, the exponentiated betas can be interpreted as percentage difference in mean values. For the choice model, the exponentiated betas are equivalent to relative odds ratios.

## Results

Table 1 show the characteristics of the study population, presented separately for male and female participants. No gender differences were observed in any of the listed characteristics. Besides non-response, there were no sources of missing data; all participants answered all questions, and none chose to withdraw from the study.

**Table 1:** Study characteristics of male and female participants in the age group 18 to 67 years (*n* = 574)

Characteristics	Male (*n* = 262)	Female (*n* = 312)	Χ^2^, *p*-value
Age (years), Mean (sd)	44.8 (13.2)	45.0 (13.5)	
Age groups, n (%)			0.3, *p* = 0.86
18–35 years	69 (26.3)	88 (28.2)	
36–50 years	85 (32.4)	101 (32.4)	
51–67 years	108 (41.2)	123 (39.4)	
Education, n (%)			1.9, *p* = 0.38
< 10 years	26 (9.9)	36 (11.5)	
10–12 years	105 (40.1)	138 (44.2)	
≥ 13 years	131 (50.0)	138 (44.2)	
Marital status, n (%)			0.2, *p* = 0.64
Married/cohabitant	120 (45.8)	149 (47.8)	
Other	142 (54.2)	163 (52.2)	
Municipal income level, n (%)			2.1, *p* = 0.34
Low	34 (13.0)	44 (14.1)	
Middle	136 (51.9)	143 (45.8)	
High	92 (35.1)	125 (40.1)	
Severity of VI, n (%)			1.4, *p* = 0.24
Moderate	77 (29.4)	106 (34.0)	
Severe or blind	185 (70.6)	206 (66.0)	
Age of VI onset, n (%)			1.8, *p* = 0.41
Congenital	124 (47.3)	161 (51.6)	
Childhood (1–24 years)	59 (22.5)	57 (18.3)	
Adulthood (≥ 25 years)	79 (30.2)	94 (30.1)	
Having other impairments, n (%)			1.7, *p* = 0.19
No	184 (70.2)	203 (65.1)	
Yes	78 (29.8)	109 (34.9)	

*VI* visual impairment, *sd* standard deviation

### Work status

Altogether, 44.1 percent of the participants were employed, more males than females (*p* = 0.002). Table 2 shows the work status of males and females from the visual impairment population relative to the general population. Compared to the general population, males and females with visual impairment had lower rates of employment and they were more often receiving disability benefits. No other differences were observed between the two populations.

**Table 2:** Work status of male and female participants with visual impairment in the age group 18 to 67 years (*n* = 574) compared with the Norwegian general population

Work status	Visual impairment n (%)	General population^b^ n (%)	Difference	*p*-value*
Males				
Employed	134 (51.2)	1314 (79.2)	− 28.0	< 0.001
Full-time	82 (31.3)	1059 (63.8)	− 32.5	< 0.001
Part-time	42 (16.0)	145 (8.7)	7.3	0.001
Self-employed	10 (3.8)	108 (6.5)	− 2.7	0.09
Unemployed	10 (3.8)	57 (4.1)	− 0.3	0.74
Outside the work force	118 (45.0)	288 (17.4)	27.6	< 0.001
Disability benefits	88 (33.6)	128 (7.7)	25.9	< 0.001
Other^a^	30 (11.5)	160 (9.6)	1.9	0.37
Females				
Employed	119 (38.1)	1184 (74.8)	− 36.7	< 0.001
Full-time	55 (17.6)	741 (46.8)	− 29.2	< 0.001
Part-time	59 (18.9)	387 (24.5)	− 5.6	0.07
Self-employed	5 (1.6)	51 (3.2)	− 1.6	0.11
Unemployed	10 (3.2)	40 (3.3)	− 0.1	0.46
Outside the work force	183 (58.7)	358 (22.6)	36.1	< 0.001
Disability benefits	136 (43.6)	169 (10.7)	32.9	< 0.001
Other^a^	47 (15.1)	189 (12.0)	3.1	0.14

^a^ participants under education, early retirement, home workers, or other statuses not encompassed by any of the listed categories; ^b^ numbers are weighted to represent the total working-age population in Norway, and presented per 1000 persons; * *p*-value derived from Pearson’s chi-squared tests. Data from the general population are available on the following web-sites: https://www.ssb.no/statbank/table/05111/tableViewLayout1/; https://www.ssb.no/statbank/table/11132/tableViewLayout1/;

https://www.ssb.no/statbank/table/11755/tableViewLayout1/

### Factors associated with employment

Results from regression analyses of sociodemographic and vision-related factors associated with employment in people with visual impairment are shown in Table 3. In unadjusted analyses, all factors, except severity of vision loss, were statistically significantly associated with employment. In the adjusted models, higher employment rates were observed among those being middle aged, male gender, higher education, and residing in high-income municipalities. Higher onset-age of vision loss, being blind or severely impaired, and having other additional impairments were associated with lower employment.

**Table 3:** Sociodemographic and vision-related factors associated with the probability of employment among working-age people with visual impairment (*n* = 574), estimated using regression analysis

Variables	Unadjusted PR (95% CI)	*p*-value	Adjusted^b^ PR (95% CI)	*p*-value
Age groups (ref. 18–35 years)				
36–50 years	1.30 (1.04, 1.62)	0.02	1.29 (1.05, 1.59)	0.02
51–67 years	0.84 (0.66, 1.08)	0.18	0.93 (0.73, 1.17)	0.53
Male sex (ref. female)	1.34 (1.12, 1.61)	0.002	1.29 (1.09, 1.53)	0.003
Education (ref. < 13 years)	2.09 (1.71, 2.55)	< 0.001	1.89 (1.56, 2.29)	< 0.001
Municipal income level (ref. low)				
Middle	1.37 (0.95, 1.99)	0.09	1.34 (0.98, 1.84)	0.07
High	1.83 (1.27, 2.63)	0.001	1.56 (1.14, 2.13)	0.006
Blind/severe VI (ref. moderate VI)	0.91 (0.75, 1.10)	0.33	0.80 (0.66, 0.96)	0.02
Age of VI onset (cont., 10 years)^a^	0.92 (0.86, 0.97)	0.002	0.91 (0.86, 0.97)	0.002
Having other impairments (ref. no)	0.52 (0.41, 0.67)	< 0.001	0.61 (0.48, 0.78)	< 0.001

*VI* visual impairment, *cont* continuous, *vs* versus, *PR* probability ratio, *CI* confidence interval; ^a^ higher scores indicates higher onset-age of vision loss; ^﻿b^ Adjusted for all variables included in the table

### Depression and life satisfaction

Table 4 shows results from the analyses of employment with outcomes of depression and life satisfaction among people with visual impairment. Additionally, the results from the full model, including covariates, are presented in online supplement (Table S1). In unadjusted analyses, employed participants had on average 30% lower score on depression compared to others. They also had a 1.97 greater odds of being in a higher category on the life satisfaction scale. After adjusting for age, gender, education, marital status, municipality income levels, and all indicated characteristics of vision loss, the strength of the associations was weakened but remained statistically significantly different for both depression (Adjusted exponentiated beta: 0.80, 95% CI: 0.67, 0.96) and life satisfaction (Adjusted exponentiated beta (odds ratio): 1.85, 95% CI: 1.32, 2.59) (Table 4).

**Table 4:** Associations of employment with outcomes of depression and life satisfaction in people with visual impairment (*N* = 574), estimated using gamma Generalized Linear Models for depression and Heterogeneous Choice Models for life satisfaction

	**Mean (SD)**	**Unadjusted Exp(beta) (95% CI)**^**a**^^**b**^	***p*-value**	**Adjusted Exp(beta) (95% CI)**^**a**^^**bc**^	***p*-value**
**Depression**					
Not employed	6.5 (6.1)	Reference		Reference	
Employed	4.6 (4.7)	0.70 (0.60, 0.83)	< 0.001	0.80 (0.67, 0.96)	0.02
**Life satisfaction**					
Not employed	6.4 (2.2)	Reference		Reference	
Employed	7.3 (1.7)	1.97 (1.52, 2.56)	< 0.001	1.85 (1.32, 2.59)	< 0.001

*Exp* exponentiated, *CI* confidence interval; ^a^ the exponentiated betas for depression can be interpreted as percentage difference in mean scores, whereas the exponentiated betas for life satisfaction can be interpreted as odds ratios; ^b^ higher scores on depression indicates more depressive symptoms, whereas higher scores on life satisfaction indicates higher life satisfaction; ^c ^adjusted for gender, age (years: 18–35, 36–50, 51–67), education (years: < 13, ≥ 13), marital status (married/cohabitant, other), municipal income level (low, moderate, high), onset-age of vision loss (continuous), severity of vision loss (moderate, severe/blind), and having other impairments (no, yes)

## Discussion

In this cross-sectional study of people with visual impairment a smaller proportion were employed (44%) compared with the general population (77%). Being of middle age, male gender, having higher education, and residing in high-income municipalities were associated with higher employment, whereas higher onset-age of vision loss, being blind or severely impaired, and having other additional impairments were associated with lower employment. Employed participants had lower levels of depression compared to others. They also had a higher odds of higher life satisfaction.

### Interpretation and comparison with other studies

The 44 percent work participation in our study is relatively high in a global context [3–6]. However, there is still a substantial difference in the employment opportunities between people with visual impairment and the general population. The observed employment gap (33%) in the present study was in the middle range compared to that reported in other Westernized countries, with a gap ranging from 23 percent in the United Kingdom [3] to 40–57 percent in the United States [3, 6].

The observed employment gap could be due to the vision loss itself, or due to poorer health among people with visual impairment [33]. In our study, most non-employed people with visual impairment reported disability benefits. Becoming recipient of disability benefits is not only a matter of health. It is a complete evaluation of people’s health and their opportunities for being included in the labour market. In situations where there are few available jobs, visual impairment in itself could be a sufficient reason for being granted disability benefits [20]. However, studies have shown that people with visual impairment have more co-morbid conditions, such as cardiovascular disorders, diabetes, and mental disorders such as depression, relative to the general population [17, 34].

The lower employment rates with increasing onset-age of vision loss are interesting, because the majority of people losing their vision during adulthood are likely to be employed when the vision loss occurred [11]. A plausible explanation for the poor employment outcomes of people with late-life vision loss could be due to challenges adapting to the vision loss [35], as well as problems accommodating the job to the vision loss or difficulties changing jobs once the carrier has been established [36]. The greater the severity of impairment, the more difficult it may be to adapt to the vision loss. Alternatively, it may also be associated with adversity in mental health, as loosing vision later in life is associated with depression [17] which is generally a strong predictor of non-employment [37].

In the present study, the strong association between having additional impairments and lower work participation illustrates that having additional disorders or disabilities may increase the risk of not being employed [38] – or the other way around, people who becomes unemployed or falls outside the labour force may be more susceptible of developing adverse health conditions [37, 38].

Our results of an association between education and higher employment rates agrees to that reported in previous studies [11–13], illustrating the importance of higher education to promote work participation. Additionally, the higher employment rates in males relative to females and younger versus older adults reflect trends observed for the general Norwegian population [23]. A similar interpretation could be made for municipal income level, as wealthy areas tends to have better access to jobs than poor ones.

The findings of employment being associated with lower levels of depression agree with that observed in studies from the general population [14]. The relationship could be bidirectional. Work is an important domain in life for most people, and usually a source of health and mental health benefits [39]. On the other hand, good mental health is a strong predictor of employment [33]. In our study, both explanations may be applicable.

### Strengths and limitations

Strengths of this study include a large nationwide random sample of people who have been diagnosed with visual impairment, the use of telephone interviews to include individuals who would not otherwise respond, and the oversampling of young adults which made us able to obtain robust data for the entire population of people with visual impairment, including those with congenital and early-life vision loss.

The study had certain limitations. First, this study relied on cross-sectional data, which restricted our ability to make any causal inferences, and, although we controlled for some potentially confounding factors, we cannot rule out the possibility of residual confounding. We tried to handle the cause and effect issue by selecting the variables that had the greatest likelihood of occurring prior to employment in time. Second, our data were based on self-reports, being susceptible to recall bias and self-desirability bias. However, self-reports is shown to be a reliable way to assess people’s current employment status relative to registry data [40], and we believe the risk of misclassification is low. Third, we had limited information about the non-responders, and do not know how non-responding might have influenced our results. Fourth and lastly, our sample was recruited from a member organization for the blind and partially sighted, which may question the representativeness of the participants. Although our sample had higher education, the employment rates in our study were the same as to that presented in census data from Statistics Norway that evaluates physical or sensory impairments via self-reports (44% versus 43%) [41].

### Implications

Employment are not only important for independent living, but also for the mental health and quality of life of people with visual impairment. A lower employment rate in people with visual impairment versus the general population may reflect barriers in the employment process such as health adversities, self-stigma, low availability of relevant jobs, employer’s negative attitudes, and transport challenges [42]. To promote participation, the labor market should be adapted in such a way that enables people with visual impairment to be included. Emphasis should be directed towards universal design, appropriate adaption and modification of the workplace [36], changing negative public attitudes towards visual impairment and disability in general [37, 42], and ensuring access to educational institutions, transportation, work places, and assistive technologies and other support services [42].

Our study showed a much narrower gap between the visual impairment population and the general population in part-time employment than that observed for full-time or self-employment. In accordance with Norwegian law [43], people with visual impairment can work part-time and receive financial compensation for the hours they are unable to work. This enables people with visual impairment to become a part of the labor force and at the same time be compensated for the fact that more time can be spent traveling to and from work, preparing for work, and performing other necessary tasks in everyday life. The opportunity to combine disability benefits and wages is one of several strategies that could create a more inclusive working life for people with physical or sensory impairments.

## Conclusions

Despite a high rate of employment among people with visual impairment in Norway (44%), there is still a significant employment gap relative to the general population, with a difference of 33 percent. Being of middle age, male gender, having higher education, and residing in high-income municipalities were associated with higher employment, whereas higher onset-age of vision loss, being blind or severely impaired, and having other additional impairments were associated with lower employment. We also found that employed participants had lower levels of depression compared to others, and were more satisfied with their life in general. Better adaption of the labor market towards people with visual impairment, such as universal design of work environments and combining part-time work and disability benefits, may promote work inclusion for these people.

## Supplementary Information

**Additional file 1: Table S1. **The entire fully adjusted model of employment with outcomes of depression and life satisfaction among working-age people with visual impairment (*N* = 574), estimated using regression analyses.

## Data Availability

Data are from the research project European Network for Psychosocial Crisis Management – Assisting Disabled in Case of Disaster (EUNAD). According to the approval from the Norwegian Regional Ethical Committee, data is to be stored properly and in line with the Norwegian privacy protection laws. Data contains sensitive information from a small group. Public availability may result in the possibility of indirect identification, and thus would compromise privacy of the participants. However, the data is freely available to interested researchers upon request, pending ethical approval from our ethical committee: post@helseforskning.etikk.no. The project leader, Prof. Trond Heir (trond.heir@medisin.uio.no), may also be contacted with requests for the data underlying our findings.
