# Supplementary material for: Visual impairment and employment in Norway
Source: BMC Public Health. 2022 Apr 5;22:648. doi: 10.1186/s12889-022-13077-0 (PMC8981612; doi:10.1186/s12889-022-13077-0)
Supplement: Supplementary file 1 — Additional file 1: Table S1. The entire fully adjusted model of employment with outcomes of depression and life satisfaction among working-age people with visual impairment (N = 574), estimated using regression analyses. [file 12889_2022_13077_MOESM1_ESM.docx]

**Online supplement**

***Depression and life satisfaction***

A description of the entire model, including covariates, of employment and its associations with depression and life satisfaction.

| **Table S1.** The entire fully adjusted model of employment with outcomes of depression and life satisfaction among working-age people with visual impairment (N = 574), estimated using regression analyses. | | | | |
| --- | --- | --- | --- | --- |
|  | **Depression** |  | **Life satisfaction** |  |
|  | **Adjusted Exp(beta) (95% CI)#§** | **p-value** | **Adjusted Exp(beta) (95% CI)#§** | **p-value** |
| **Main factor** |  |  |  |  |
| Employment (ref. no) | 0.80 (0.67, 0.96) | 0.016 | 1.85 (1.32, 2.59) | < 0.001 |
| **Covariates** |  |  |  |  |
| Age groups (ref. 18–35 years) |  |  |  |  |
| 36–50 years | 1.11 (0.91, 1.35) | 0.32 | 1.18 (1.06, 1.32) | 0.003 |
| 51–67 years | 0.66 (0.54, 0.81) | < 0.001 | 0.75 (0.59, 0.85) | < 0.001 |
| Male sex (ref. female) | 0.78 (0.68, 0.91) | 0.002 | 0.95 (0.76, 1.20) | 0.68 |
| Education (ref. < 13 years) | 0.93 (0.79, 1.09) | 0.39 | 0.92 (0.71, 1.18) | 0.50 |
| Married/cohabitant (ref. no) | 0.87 (0.74, 1.02) | 0.09 | 1.71 (1.29, 2.26) | < 0.001 |
| Municipal income level (ref. low) |  |  |  |  |
| Middle | 0.99 (0.79, 1.09) | 0.91 | 0.80 (0.62, 1.02) | 0.07 |
| High | 1.15 (0.90, 1.47) | 0.25 | 0.98 (0.60, 1.60) | 0.93 |
| Blind/severe VI (ref. moderate VI) | 1.10 (0.92, 1.31) | 0.31 | 1.04 (0.81, 1.34) | 0.75 |
| Age of VI onset (cont., 10 years) | 1.10 (1.04, 1.15) | < 0.001 | 0.90 (0.83, 0.97) | 0.006 |
| Having other impairments (ref. no) | 1.58 (1.34, 1.85) | < 0.001 | 0.65 (0,49, 0.86) | 0.003 |

*Note.* VI: visual impairment; cont: continuous; vs: versus; exp.: exponentiated; CI: confidence interval. #: The exponential betas for depression can be interpreted as percentage difference in mean scores, whereas the exponential betas for life satisfaction can be interpreted as odds ratios; §: higher scores on depression indicates more depressive symptoms, whereas higher scores on life satisfaction indicates higher life satisfaction.
